# Cross-Talk of Focal Adhesion-Related Gene Defines Prognosis and the Immune Microenvironment in Gastric Cancer

**DOI:** 10.3389/fcell.2021.716461

**Published:** 2021-10-01

**Authors:** Deli Mao, Rui Xu, Hengxing Chen, Xiancong Chen, Dongsheng Li, Shenglei Song, Yulong He, Zhewei Wei, Changhua Zhang

**Affiliations:** ^1^Digestive Diseases Center, The Seventh Affiliated Hospital of Sun Yat-sen University, Shenzhen, China; ^2^Guangdong Provincial Key Laboratory of Digestive Cancer Research, The Seventh Affiliated Hospital of Sun Yat-sen University, Shenzhen, China; ^3^Department of Gastrointestinal Surgery, The First Affiliated Hospital of Sun Yat-sen University, Guangzhou, China

**Keywords:** gastric cancer, focal adhesion, immune microenvironment, prognostic signature, biomarker

## Abstract

**Background:** Focal adhesion, as the intermediary between tumor cells and extracellular matrix communication, plays a variety of roles in tumor invasion, migration, and drug resistance. However, the potential role of focal adhesion-related genes in the microenvironment, immune cell infiltration, and drug sensitivity of gastric cancer (GC) has not yet been revealed.

**Methods:** The genetic and transcriptional perspectives of focal adhesion-related genes were systematically analyzed. From a genetic perspective, the focal adhesion index (FAI) was constructed based on 18 prognosis-related focus adhesion-related genes to evaluate the immune microenvironment and drug sensitivity. Then three prognosis-related genes were used for consistent clustering to identify GC subtypes. Finally, use FLT1, EGF, COL5A2, and M2 macrophages to develop risk signatures, and establish a nomogram together with clinicopathological characteristics.

**Results:** Mutations in the focal adhesion-related gene affect the survival time and clinical characteristics of GC patients. FAI has been associated with a shorter survival time, immune signaling pathways, M2 macrophage infiltration, epithelial-mesenchymal transition (EMT) signaling, and diffuse type of GC. FAI recognizes ALK, cell cycle, and BMX signaling pathways inhibitors as sensitive agents for the treatment of GC. FLT1, EGF, and COL5A2 may distinguish GC subtypes. The established risk signature is of great significance to the prognostic evaluation of GC based on FLT1, EGF, and COL5A2 and M2 macrophage expression.

**Conclusion:** The focal adhesion-related gene is a potential biomarker for the evaluation of the immune microenvironment and prognosis. This work emphasizes the potential impact of the focal adhesion pathway in GC therapy and highlights its guiding role in prognostic evaluation.

## Introduction

Gastric cancer (GC) is a highly heterogeneous tumor. The death of GC patients every year brings a huge burden to the global economy. The 2019 survey showed that GC deaths accounted for 8% of all patients (Ferlay et al., 2019). The view that the interaction of multiple genes is an important promoter of tumor progression has been continuously accepted by the public. For this reason, many prognostic models have been established in recent years in an attempt to accurately evaluate prognosis and treatment. Zhang et al. (2020) revealed the potential role of N6-methyladenosine (m6A) modification in the microenvironment of GC from the perspective of epigenetics. Using transcriptomics of long non-coding RNA (lncRNA) to explore a new GC subtype with prognostic value (Chen et al., 2021). In addition, a proteomics signature has been developed to improve the diagnostic ability of GC (Song et al., 2021). Due to the individual differences and complexity of the pathogenesis of GC, the results of a single omics prognosis model are not satisfactory from the perspective of multiple omics, which forces people to explore new perspective models to accurately describe the prognosis of GC.

Previous studies have shown that GC is driven by a variety of key signaling pathways (Molaei et al., 2018). Focal adhesion kinase (FAK) is a kind of cytoplasmic non-receptor protein tyrosine kinase, which regulates tumor invasion, movement, and survival (Lee et al., 2015). FAK activates Ras (Fu et al., 2017), PI3K (Guo et al., 2020), ERK1/2 (Salgado-Lucio et al., 2020) by transmitting extracellular signaling from integrins, growth factors, and mechanical stimuli to cells, finally causing tumor adhesion and migration. The combination of extracellular matrix (ECM) and integrin will recruit FAK to the position where integrin gathers, which is called “focal adhesion”. The focal adhesion signaling pathway has been proved to have a great influence on the regulation of ECM cell migration (Zhou et al., 2021) and tumor microenvironment (Murphy et al., 2020). Molecular crosstalk in the focal adhesion pathway is a key factor in mediating tumor-ECM interactions (Eke and Cordes, 2015). Cytokines secreted by cancer-related fibroblasts derived from ECM in GC activate the β1 integrin-FAK-YAP signaling axis to induce drug resistance of tumors (Uchihara et al., 2020). CXCL1 is secreted by lymphatic endothelial cells in the tumor microenvironment and promotes the invasion, migration and adhesion of GC cells by activating integrin β1-FAK-AKT signaling (Wang et al., 2017). Immune evasion is a complex problem in tumor immunotherapy. Interference of small molecule inhibitor VS-4718 with focal adhesion pathway induces tumor regression and enhances antitumor immunity (Serrels et al., 2015). FAK Improves expression of inflammatory factors IL33 and SST2 in rat squamous cell carcinoma and inhibits CD8^+^T cell-mediated antitumor effect (Serrels et al., 2017). Adjuvant chemotherapy for breast cancer targeting FAK can reduce macrophage infiltration and tumor growth (Wendt and Schiemann, 2009). Focal adhesion pathway is the bridge between tumor and ECM. However, there is currently no focal adhesion-related gene signature to evaluate the prognosis of GC, and which is expected to become a new target for evaluating the immune microenvironment and prognosis of GC.

In this study, we explored genomic changes in 875 GC samples based on The Cancer Genome Atlas (TCGA) and gene expression omnibus (GEO) database. It is found that the mutation of genes related to the focal adhesion pathway significantly affects the prognosis of GC patients. Focal adhesion gene expression may identify GC prognostic subtypes. Based on the expression of the focal adhesion-related gene, we calculated the focal adhesion index (FAI), which is not only related to M2 macrophage cell infiltration, but also significantly related to microsatellite instability (MSI), tumor mutation burden (TMB), and epithelial-mesenchymal transition (EMT). Finally, we established a focal adhesion signature and verified its guiding significance in GC prognostic evaluation.

## Materials and Methods

### Data Acquisition and Processing

The Cancer Genome Atlas-STAD somatic mutation data, RNA-seq profile (FPKM value), and corresponding GC patient clinical information were obtained from TCGA website^1^. Expression data and clinical information for both GEO cohorts including GSE66229 and GSE15459 were downloaded from the Gene-Expression Omnibus (GEO) Database^2^. The FPKM value of the TCGA dataset was converted into transcripts per kilobase million (TPM) values. The microarray data of the GEO datasets were background corrected and normalized by “simpleaffy” and “affy” packages. The “sva” package is used to correct batch effect based on the ComBat method (Leek et al., 2012). The mRNA expression-based stemness index (mRNAsi) of GC was reported by Malta et al. (2018). TMB of GC was extracted according to the method we reported before (Mao et al., 2021). The focal adhesion-related genes (Supplementary Table 1) were obtained from c2.cp.kegg.v7.2.symbols of the Molecular Signatures Database (Msigdb)^3^. In this study, TCGA data were divided into training datasets and GEO data were classified as validation datasets.

### Functional Enrichment Analysis

The DAVID online functional annotation website^4^ was adopted for prognostic-related somatic mutation gene KEGG enrichment analysis. The analysis results are visualized using the R package “ggplot2”. To study the signaling difference of participation under different FAI subtypes, the “GSVA” R package (Hanzelmann et al., 2013) was applied to analyze Gene Set Variation Analysis (GSVA) based on h.all.v7.2 gene set. Difference analysis was performed using the “limma” package for analysis results, and the “pheatmap” package was used for visual heatmap drawing. Gene Set Enrichment Analysis (GSEA) was performed *via* GSEA software (Subramanian et al., 2007). FDR <0.25 was considered statistically significant.

### Evaluation of Immune Cell Infiltration Abundance and Calculation of the Immune Score

The CIBERSORT algorithm was used to assess the abundance of infiltration of 22 immune cells based on gene expression microarray^5^ (Becht et al., 2016), which includes immune cells of different functional states and cell types. The immune score of the samples was calculated using the R-package “estimate” according to the mRNA expression matrix, which represents the total immune cell infiltration level in the tumor tissue (Yoshihara et al., 2013).

### Construction of Focal Adhesion Index and Subtype Recognition

Single Sample Gene Set Enrichment Analysis (ssGSEA) was used to evaluate sample FAI based on the transcriptome data of 18 prognostic-related somatic mutation genes using the “GSVA” package. ssGSEA score was normalized to the range of 0–1. The “survminer” package was used to find the optimal cut-off value for FAI for the best prognostic subgroup. In order to reduce that dimension, the Univariant Cox regression analysis was performed to screen for prognosis-related focal adhesion genes from a transcriptional perspective. To evaluate the ability of prognosis-related genes to recognize GC subtypes, Consensus Clustering was applied to explore subtype classification based on gene transcription level through the “ConsensusClusterPlus” package. Relevant parameter settings refer to previous research reports (Gong et al., 2020).

### Establishment of Prognostic Signature

The Least Absolute Shrinkage and Selection Operator (LASSO) cox regression algorithm is performed to construct the prognostic signature and calculate the factor weight coefficients through the R package “glmnet”. The sample riskscore is calculated according to the following formula:


$$\text{riskscore}=\sum_{i=1}^{n}C\,o\,e\,f_{i}\times X_{i}$$

*Coef*_*i*_ represented the weight coefficient of each factor, and *X*_*i*_ represented the factor expression level. The median value of riskscore of all samples of the training set was defined as the cut-off value. According to the cut-off value, the samples of the training and validation cohorts were divided into a high-risk group (greater than cut-off) and a low-risk group (less than cut-off).

### Drug Sensitivity Analysis

Genomics of Drug Sensitivity in Cancer (GDSC) (Yang et al., 2013) is a publicly available genomic database for tumor therapy, dedicated to finding potential therapeutic targets to improve tumor therapeutic efficacy. We downloaded GC cell line expression data and corresponding IC50 values^6^ for 265 drugs from the GDSC database. Samples FAI were evaluated based on transcription levels. Spearman correlation analysis was used to calculate the correlation between FAI and drug IC50. *P* < 0.05 was considered statistically significant.

### Statistical Analysis

Kaplan-Meier curve analysis is used for prognostic analysis. A Chi-square test is used to compare the clinical characteristics of mutation or non-mutation mode. The Wilcoxon test was performed to compare the differences between the two sets of data. The receiver operating characteristic (ROC) curve was tested to predict the efficiency of the overall survival rate of GC patients. Pearson’s correlation analysis was used to compare the correlation of mRNA expression levels among 18 focal adhesion-related genes. Prism 8, SPSS19.0, and R software (version 3.6) were applied for statistical analysis and graphing. *P* < 0.05 was considered statistically significant.

## Results

### Screening of Somatic Mutations Related to Prognosis

**FIGURE 1:**
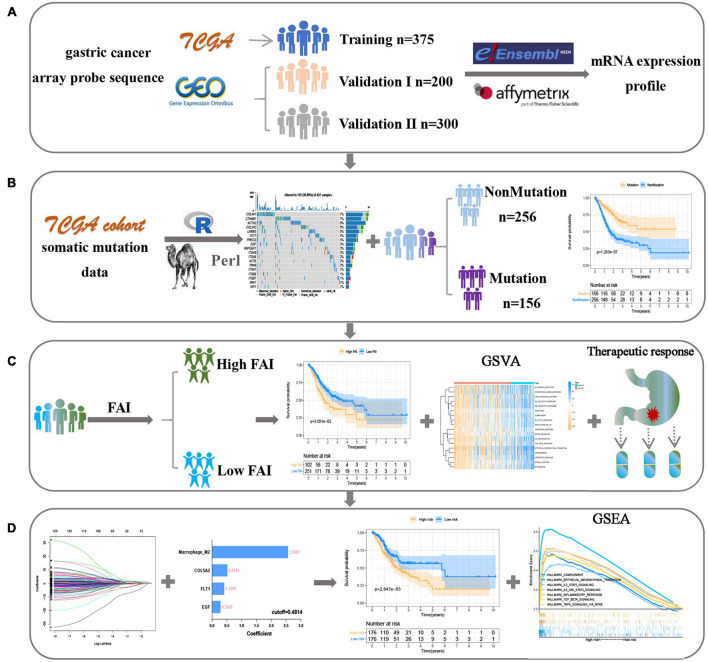
The flow chart shows the process of this work. **(A)** Data acquisition and processing. **(B)** Explore focal adhesion mutation type. **(C)** The establishment of FAI is based on the expression of focal adhesion-related genes. **(D)** Development and verification of the prognostic signature.

**FIGURE 2:**
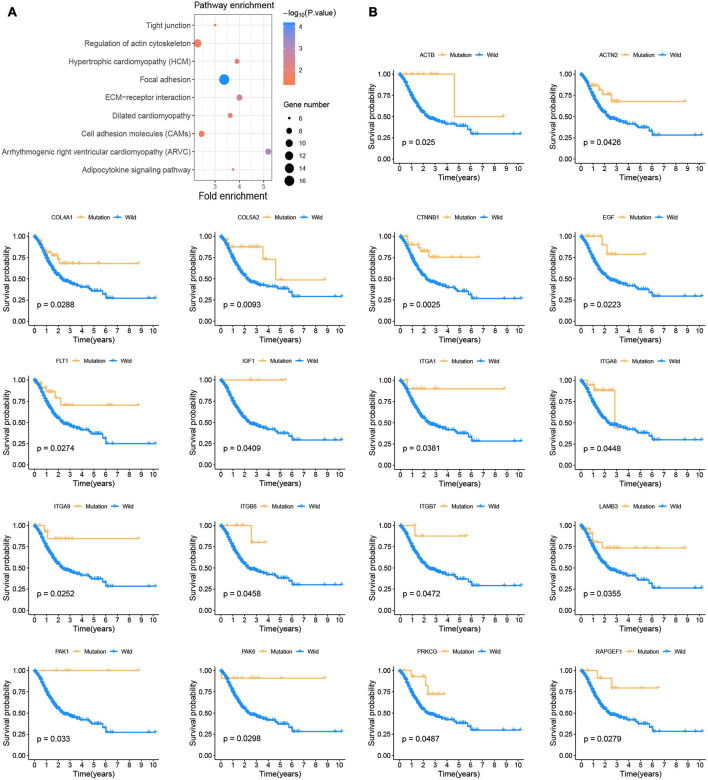
Somatic mutations affect the prognosis of patients with GC **(A)** KEGG enrichment analysis of prognostic-related somatic mutation genes. **(B)** Kaplan-Meier survival curve of 18 focal adhesion pathway-related genes.

This study was conducted following the (Figures 1A–D) process. According to the published somatic mutation data of TCGA-STAD, genes with a mutation frequency of more than 10 were subjected to prognostic analysis, and the results showed that the mutations of 416 genes were related to the prognosis of GC (Supplementary Table 2). To identify the signaling pathways involved in these prognosis-related mutated genes, we performed KEGG enrichment analysis. The focal adhesion pathway was most significantly enriched (Figure 2A). In addition to ECM-receptor interaction, cell adhesion. Therefore, we seriously suspect that somatic mutations in genes related to the focal adhesion pathway have a profound impact on patients with GC. For this reason, we investigated 18 somatic mutations in the focal adhesion pathway that are related to prognosis. Kaplan-Meier curve analysis of these 18 genes showed that the survival time of patients in the mutation group was significantly longer than that in the non-mutation group (Figure 2B).

### Focal Adhesion Mutation Type Is Closely Related to Prognosis and Clinicopathological Characteristics

**FIGURE 3:**
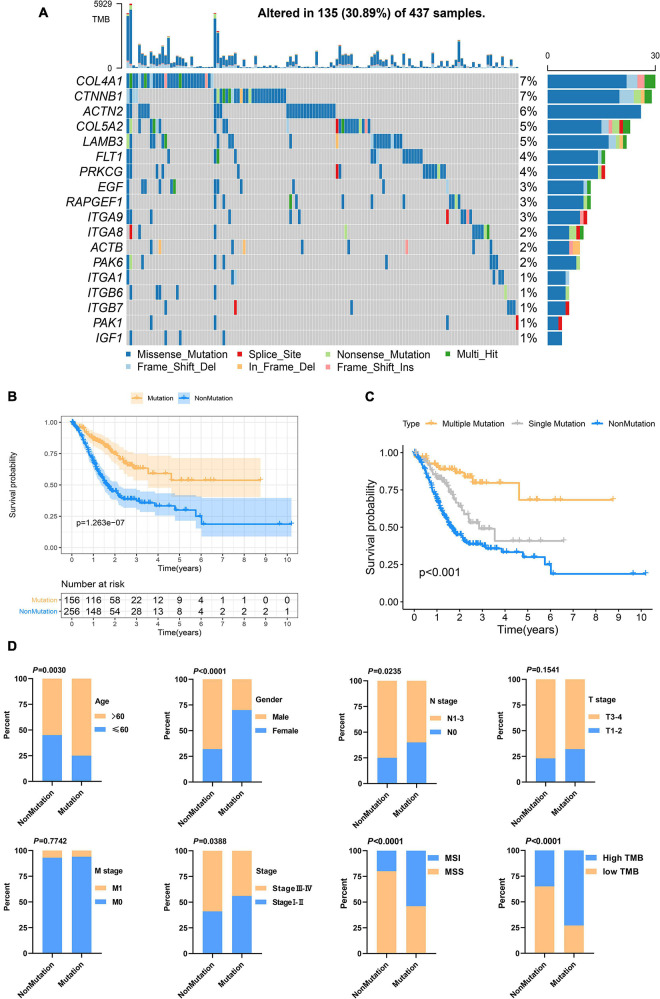
Focal adhesion mutations are closely related to the prognosis and clinicopathological characteristics of GC patients **(A)** The mutation frequency of 18 focal adhesion-related genes in 437 GC patients in the TCGA cohort. **(B)** Kaplan-Meier curve shows that the survival time of the focal adhesion mutation group of GC patients in the TCGA cohort is significantly longer than that of the non-mutation group. **(C)** The overall survival of GC patients under different mutation types. **(D)** Chi-square test shows that the focal adhesion mutation is closely related to the clinicopathological characteristics.

Genetic alteration in the prognosis-related focal adhesion gene was explored in GC to assess the frequency of somatic mutations in 18 genes. In the TCGA-STAD cohort, each of the focal adhesion genes had a low mutation rate (Figure 3A), COL4A1 and CTNNB1 had the highest mutation rate (7%) followed by ACTN2 (6%), and ITGA1, ITGB6, ITGB7, PAK1, and IGF1 had the lowest mutation rate. To observe the overall mutation pattern of these genes, samples with focal adhesion mutations were considered to be mutation samples. Therefore, the samples were divided into mutation groups and non-mutation groups. We found that the survival time of GC patients in the mutation group was significantly longer than that in the non-mutation group (*P* = 1.263E^–7^) (Figure 3B). To more accurately reflect the relationship between mutation efficiency and prognosis, we divided GC patients into the non-mutation group, single gene mutation group, and multiple gene mutation groups. We found that the survival time of the multi-mutation group was longer than that of the single mutation group, and that of the single mutation group was longer than that of the non-mutation group (*P* < 0.001) (Figure 3C). This result suggested that focal adhesion acted as an oncogene pathway, and these genes mainly underwent missense mutation, which might cause structural changes of proteins and loss of functions, thus losing the main functions of focal adhesion. Subsequently, the association of the focal adhesion mutation with the clinicopathological characteristics of GC patients was analyzed (Figure 3D). We found that the mutations were the result of time accumulation, and the proportion of patients older than 60 years in the mutant group was significantly higher than that in the non-mutant group (*P* = 0.003). At the same time, the number of women in the mutant group was significantly higher than that of men (*P* < 0.001), while the number of men in the non-mutant group was higher than that of women. In addition, the N stage (*P* = 0.0235) and stage (*P* = 0.0388) were also closely related to the mutation state. In recent years, Gurzu et al. (2017) proposed a new Dukes-MAC-like staging system to classify GC subtypes, which was defined according to the T and N stage. Probably because T staging (*P* = 0.1541) was not significantly different from the mutant group, the Dukes-MAC-like stage system was not significantly different between the mutant and non-mutant groups (*P* = 0.3036) (Supplementary Figure 1A). Next, we explored and found that the mutation group was more likely to cause microsatellite instability and higher tumor mutation load. It has been reported in the previous study that the tumor stem cell index is an important indicator to drive tumor progress (Malta et al., 2018). Here, whether there is an association between the focal adhesion mutation and mRNAsi, our results show that the mRNAsi of patients with mutations is significantly higher than that of patients without mutations (*P* < 0.0001) (Supplementary Figure 1B). The previous study (Mao et al., 2021) has reported that patients with high mRNAsi have a better prognosis. In terms of prognosis, the results of this study are consistent with them. In summary, focal adhesion-related gene mutation in GC patients is closely related to the prognosis and clinical characteristics.

### Genetic and Transcriptional Alteration of Focal Adhesion-Related Genes

**FIGURE 4:**
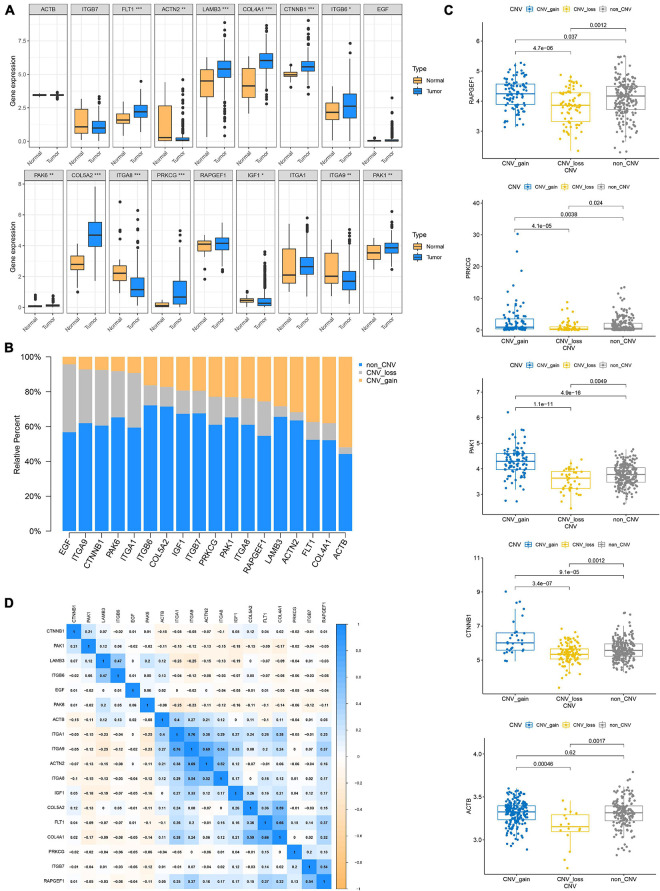
Genetic and transcriptional alteration of focal adhesion-related genes. **(A)** The expression of genes related to focal adhesion is different between normal and tumor tissues, *P < 0.05; **P < 0.01; ***P < 0.001. **(B)** CNV again, CNV loss, and non-CNV frequency of focal adhesion-related genes in the TCGA cohort. **(C)** Correlation between CNV alteration of focal adhesion-related genes and gene expression. **(D)** Correlation between expression levels of focal adhesion-related genes.

Since genetic alterations are associated with the prognosis of patients with GC, does it affect gene expression? First, we compared the expression of these genes in normal and tumor tissues and found that most of the genes were up-regulated in the tumor (Figure 4A). Somatic copy number changes of focal adhesion-related genes were then examined (Figure 4B). ACTB, COL4A1, FLT1, and ACTN2 were found to have abundant CNV gain. EGF, ITA9, CTnNB1, and PAK6 have extensive CNV loss. Next, the relationship between the expression level of 18 genes and CNV were analyzed. The expression levels of PAPGEF1, PRKCG, PAK1, and CTNNB1 were higher than that of non-CNV on CNV gain and lower than that of non-CNV on CNV loss. ACTB expression level was lower than that of non-CNV on CNV loss, but CNV gain was not higher than that of non-CNV (Figure 4C). CNV gain drives the increase of gene expression in tumors, while CNV loss causes the decrease of gene expression. However, this complex mechanism cannot completely explain the change of expression level of all focal adhesion-related genes (Supplementary Figure 2). Finally, based on transcriptome level, we evaluated the expression correlations of 18 genes and found that most of them exhibited positive correlations (Figure 4D), especially the expressions of ITGA 1, ITG A9, ACT N2, ITG A8, IFG 1, Col 5A2, FLT 2, and Col 4A1, suggesting that there was crosstalk between focal adhesion-related genes, which was important for GC progression.

### Focal Adhesion Index Construction

**FIGURE 5:**
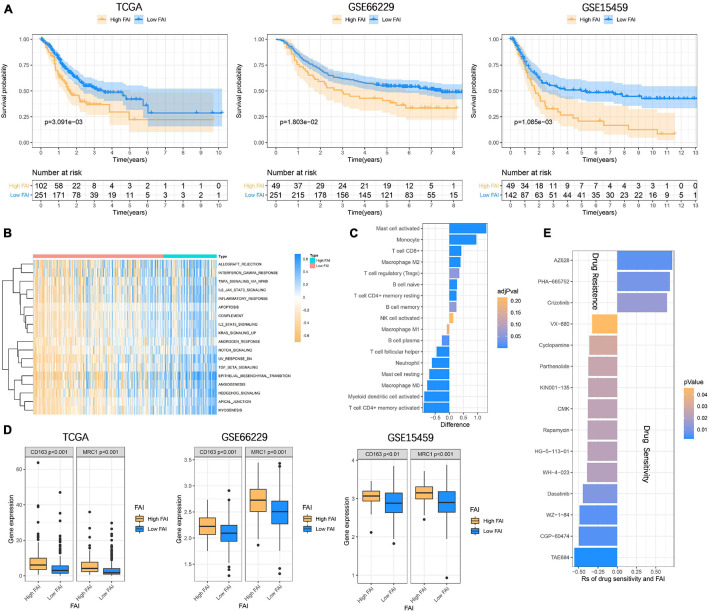
Focal adhesion index assesses the prognosis and immune cell infiltration **(A)** FAI is closely related to the overall survival of GC patients, and the survival time of High FAI patients is significantly lower than that of Low FAI. **(B)** The heatmap shows that the immune signaling pathway is significantly enriched in the High FAI group. **(C)** The immune cells infiltrated in the high and low FAI groups. **(D)** M2 macrophage markers expression. **(E)** Major signaling pathway inhibitor explorations in the GDSC database.

In order to enable focal adhesion-related genes to better evaluate the overall adhesion ability of the sample, we constructed FAI based on the expression levels of 18 genes in the TCGA cohort. The optimal cut-off value (cut-off = 0.6361) was calculated according to the steps described in the method to classify the samples as High FAI and Low FAI. The survival time of patients with high FAI in the TCGA cohort was significantly lower than that of patients with Low FAI (*P* = 3.091E^–3^). The validation cohorts also came to a consistent conclusion (GSE66229, *P* = 1.803E^–2^; GSE15459, *P* = 1.085E^–3^) (Figure 5A). GSVA was used to explore the differences in signaling pathways between Low and High FAI. The results showed that there was significant enrichment of immune-related signaling, such as Interferon-gamma response, IL6 JAK STAT3 signaling, IL2 STAT5 signaling, and TGF-β signaling. Significant enrichment of epithelial-mesenchymal transition (EMT) signaling was also observed (Figure 5B). This result suggested that FAI was closely related to tumor immunity and tumor microenvironment. Next, we analyzed whether FAI was related to the immune score. It was found that the immune score of high FAI samples was correspondingly increased in both the training (*P* < 0.0001) and the validation groups (GSE66229, *P* < 0.0001; GSE15459, *P* = 0.0005) (Supplementary Figure 3A). Cumulative evidence indicates the relationship between focal adhesion and the immune microenvironment. Different immunocyte infiltration was specifically analyzed to assess the effect of FAI on the immune microenvironment. The levels of CD8^+^T cells, M2 macrophage, and regulatory T cells (Tregs) at high FAI were significantly increased, while the levels of macrophage M0, T cell CD4^+^ memory activated, and myeloid dendritic cell activated were significantly decreased (Figure 5C). M2 macrophage markers were then compared at low-high FAI. Consistent with previous results, these markers were significantly up-regulated in the High FAI group (*P* < 0.01) (Figure 5D). Subsequently, significant differences between low-high FAI were observed for MSI and TMB. The Low FAI group corresponded to MSI (*P* = 0.0061) and high TMB (*P* = 0.0006) (Supplementary Figure 3B). The previous GSVA enriched the EMT signaling among the low-high FAI groups. Epithelial markers were found to be significantly increased in the Low FAI group, while mesenchymal markers were significantly decreased in the Low FAI group (Supplementary Figure 3C). This result is consistent with GSVA. Lauren classification is currently a widely used GC classification method in clinical applications. According to histological characteristics, it is divided into the diffuse, intestine, and mixed types (Lauren, 1965). The diffuse type generally shows resistance to chemotherapy and has a worse prognosis (Gurzu et al., 2015; Pernot et al., 2015). We found that the diffuse type has a higher FAI than the Intestine and mixed type, and there is no statistical difference between the Intestine and mixed type (Supplementary Figure 3D). In addition, we also found a statistically significant difference in the transcription of focal adhesion-related genes between low-high FAI groups (Supplementary Figure 4A). Patients with high FAI groups correspond to higher stages and stages (*P* < 0.05) (Supplementary Figure 4B). Finally, in order to further reflect the effect of FAI on the drug, the correlation was evaluated between FAI and the response to the drug in GC cell lines. 12 pairs of compounds showed sensitivity related to FAI. Including ALK inhibitor TAE684 (Rs = ^–^0.557, *P* = 0.0022); cell cycle inhibitor CGP-60474 (Rs = −0.498, *P* = 0.0052); bone marrow tyrosine kinase on chromosome X(BMX) inhibitor WZ-1- 84 (Rs = −0.49, *P* = 0.0059). 3 pairs of drug-resistant compounds are related to FAI. Including BRAF inhibitor AZ628 (Rs = 0.713, *P* = 0.0062); MET inhibitor PHA-665752 (Rs = 0.683, *P* = 0.0092); MET inhibitor Crizotinib (Rs = 0.646, *P* = 0.0151) (Figure 5E). Taken together, these results suggest that FAI can assess GC immune cell infiltration and is associated with drug susceptibility. FAI may be used as a potential biomarker to guide the treatment and effect evaluation of GC.

### Molecular Subtype Recognition of Gastric Cancer

**FIGURE 6:**
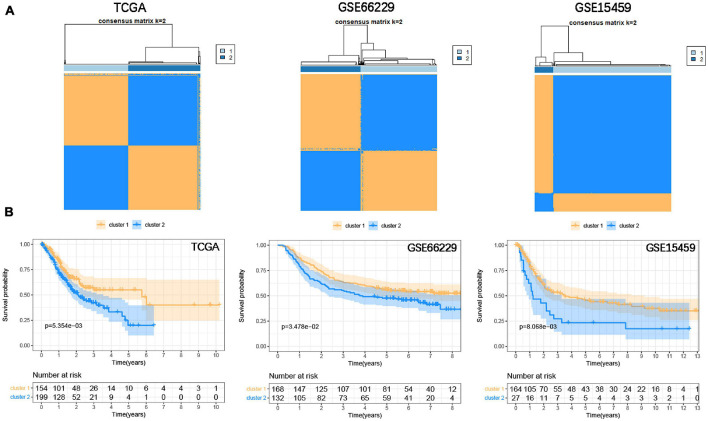
Identify the molecular subtypes of GC based on the prognostic-related focal adhesion genes. **(A)** Identify two different GC molecular subtypes in the training and validation cohorts through the consensus clustering method. **(B)** Kaplan-Meier curve is depicted to observe the relationship between molecular subtypes and the overall survival of GC patients in the training and validation cohorts.

To classify the focal adhesion status of GC samples in TCGA and GEO cohorts, the consensus clustering method was conducted to identify molecular subtypes based on the expression levels of three prognostic-related focal adhesion genes (FLT1, EGF, and COL5A2) (Supplementary Figure 4C). From the results of Supplementary Figure 5 when *K* = 2 is displayed; the relative change in the area under the CDF curve is large. When *k* = 2, the higher the intra-group correlation, the lower the correlation between groups (Figure 6A), so we divided the GC patients into two groups in the TCGA cohort, respectively, cluster1 and cluster2. Kaplan-Meier curves were drawn to evaluate the prognostic differences between clusters. The survival time of GC patients in the cluster1 group was significantly higher than that in the cluster2 group (*P* = 5.345E^–3^) and was also confirmed in the validation cohorts (GSE66229, *P* = 3.478E^–2^; GSE15459, *P* = 8.068E^–3^) (Figure 6B). The results confirmed that three prognosis-related focal adhesion genes could well distinguish the different subtypes of GC, and these molecular subtypes could significantly identify the prognosis stratification of GC.

### Development and Verification of the Prognostic Signature

**FIGURE 7:**
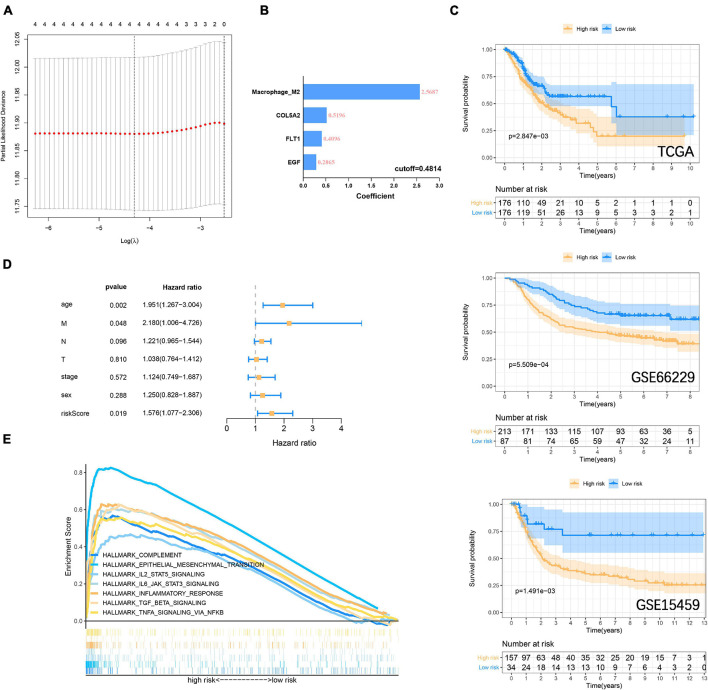
Construction and verification of risk prognostic signature **(A)** LASSO Cox regression model shows that four risk factors are included in the risk signature. **(B)** Four risk factor coefficients and cut-off values. **(C)** Kaplan-Meier curve is drawn to evaluate the overall survival of GC patients in low-high risk groups. **(D)** Riskscore and clinicopathological characteristics Multivariate cox regression analysis in the TCGA cohort. **(E)** GSEA shows that immune-related signaling pathways are significantly enriched in the high-risk group.

In the previous study, M2 macrophage was found to be closely related to the focal adhesion pathway (Ngabire et al., 2020; Li et al., 2021). In the past, it has been widely reported that M2 macrophage derived from immune microenvironment participate in the process of GC metastasis and EMT (Chen et al., 2017; Li et al., 2019). Here we incorporate the M2 macrophage together with COL5A2, FLT1, and EGF into the LASSO Cox regression model (Figure 7A), and evaluate the riskscore according to the coefficient of each variable (Figure 7B). According to the cut-off = 0.4814 in the training group, the samples were divided into high-low risk groups. The Kaplan-Meier curve revealed that the overall survival of GC patients in the high-risk group was significantly lower than that in the low-risk group in the training (*P* = 2.847E^–3^) and validation (GSE66229, *P* = 5.509E^–4^; GSE15459, *P* = 1.491E^–3^) datasets (Figure 7C). The area under the curves (AUC) for 1, 3, and 5-years overall survival were 0.701, 0.626, and 0.613, respectively, in the training set. 1, 3 and 5-years AUC in the GSE66229 dataset were 0.647, 0.617, and 0.609, respectively. 1, 3, and 5-years AUC in the GSE15459 dataset were 0.654, 0.640, and 0.613, respectively (Supplementary Figure 6A). Multivariate cox regression analysis also suggests that riskscore is a more important risk factor for GC than stage level (Figure 7D) (Supplementary Figure 6B). Finally, GSEA was carried out to explore the signaling pathways between the high-low risk groups. It was consistent with our previous GSVA results. IL6 JAK STAT3 signaling, IL2 STAT5 signaling, TGF-β signaling, and inflammatory response, and other immune signals were enriched in the high-risk group (Figure 7E).

**FIGURE 8:**
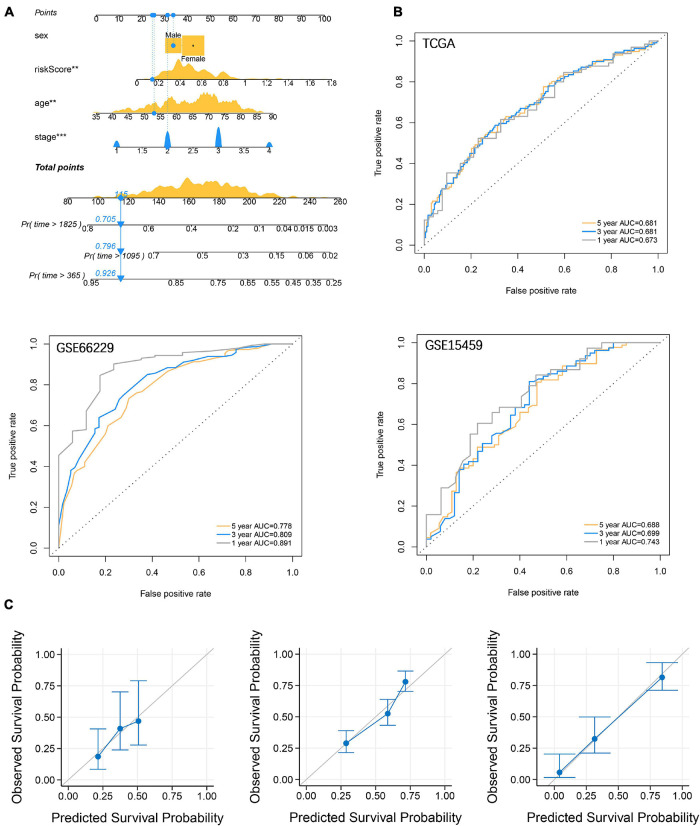
Establishment and verification of nomograms. **(A)** Riskscore and clinicopathological characteristics were used to construct nomograms **(B)** The ROC curve describes the efficiency of the nomogram for predicting the overall survival of GC 1, 3, and 5 years. **(C)** Calibration curves were used to evaluate the accuracy of nomograms in predicting the 5-year overall survival of GC patients.

To enhance the efficiency of traditional clinicopathological characteristics in predicting the 1-, 3-, and 5-year overall survival of GC. We introduce riskscore into the nomogram model to improve the accuracy of the prediction. When the total points were 115, the 1-year survival rate for GC patients was 0.926, the 3-year survival rate was 0.796, and the 5-year survival rate was 0.705 (Figure 8A). The ROC curve showed that the nomograms were efficient in predicting the 1, 3, and 5-year overall survival of GC patients in the training (AUC = 0.673, 0.681 and 0.681, respectively) and validation (GSE66229, AUC = 0.891, 0.809 and 0.778, respectively; GSE15459, AUC = 0.743, 0.699 and 0.688, respectively) cohorts (Figure 8B). Finally, the difference between the prediction probability and the observation probability of the five-year overall survival of GC patients was compared by using the calibration curve, and the result showed that the difference was low (Figure 8C), suggesting that the nomogram was more accurate in predicting the five-year overall survival of GC patients. The above results have demonstrated that the focal adhesion-related gene can be used as an important molecule in GC risk stratification, and is a key marker for GC prognosis and immune microenvironment assessment.

## Discussion

The accumulated evidence indicates that the focal adhesion pathway plays an important role in regulating cell survival, mediating tumor-ECM interaction, and proliferation, migration, and invasion. Although many studies have focused on the mechanism of a molecule in the pathway based on omics. However, there is no study on the overall molecular interaction of the focal adhesion pathway from the perspective of multi-omics. In this study, we revealed the alterations and differences in the GC of focal adhesion pathway-related molecules from the Genetic and transcriptional perspectives. Somatic accumulation of the focal adhesion gene with a low mutation rate favors a better prognosis for patients with GC. The focal adhesion genes are oncogenes that drive tumor progression, and their mutations cause structural changes and functional defects of proteins, thereby inhibiting tumor growth (Chattopadhyay et al., 2010; Malfait et al., 2010). Therefore, they are low mutation and relatively conservative in tumors. In addition, the focal adhesion mutation has also been associated with pathological characteristics, MSI, and TMB. Then, we constructed FAI based on the transcription levels of 18 focal adhesion genes to describe the strength of the focal adhesion pathway. The High FAI subtype is closely related to the short survival time of GC patients. The FAI subtype can also be used as an important reference basis for clinical Lauren classification. Our study shows that high FAI corresponds to the diffuse type of GC. GSVA showed that immune signaling such as IL6, IL2, TNFA, and EMT was activated in the high FAI subtype. The infiltration abundance of M2 macrophages and Tregs cells was significantly increased in the high FAI subtype. The M2 macrophage marker was also significantly elevated in the high FAI subtype.

Previous studies have shown that M2 macrophage is a marker of peritoneal metastasis of GC, and the interaction between M2 macrophages and tumor cells in the immune microenvironment is an important factor to promote tumor proliferation and metastasis (Yamaguchi et al., 2016). At the same time, M2 macrophage is also a marker of tumor immunotherapy and anti-angiogenesis treatment response (Gambardella et al., 2020). Inhibition of the focal adhesion pathway slows tumor infiltration by attenuating TGF-β signaling and macrophage infiltration (Wendt and Schiemann, 2009). In this study, we established FAI using the focal adhesion gene and found that it was closely related to M2 macrophage infiltration. M2 macrophage markers CD163 (Yamaguchi et al., 2016) and MRC1 (Xiao et al., 2021) were also observed to be significantly higher in the high FAI group than in the low FAI group. EMT signaling is a key factor for tumor resistance, regulation of angiogenesis, and promotion of growth (Sabbah et al., 2008; Banias et al., 2020). We observed a decrease in the epithelial marker in the high-FAI group and an increase in the mesenchymal markers. TGF-β signaling regulates tumor apoptosis, differentiation, proliferation, immune evasion, and other activities. It is also a key regulatory pathway for EMT signaling (Syed, 2016). TGF-β pathway facilitates the polarization of M2 macrophages, inactivates M1-macrophages, and inhibits T cell proliferation (Mia et al., 2014; Liu et al., 2019). Conversely, M2 macrophages also induce EMT processes through TGF-β signaling (Zhu et al., 2017). In this study, we found that TGF-β was significantly enriched in the high FAI group. The interaction among M2 macrophages, TGF-β signals, and EMT is an important biological process to promote tumor progression. Finally, we use FAI to further explore sensitive drug molecules targeting key pathways. ALK, cell cycle, and BMX inhibitors were sensitive compounds in the high FAI group. This indicates that patients with high FAI may benefit from these signaling pathways rather than the BRAF and MET signaling pathways.

Next, we used the three focal adhesion genes FLT1, EGF, and COL5A2 related to prognosis to divide the samples into two subtypes in the TCGA cohort. And it was verified in the GEO cohorts. It suggested that the transcriptomics of these three genes could be used as an important basis for prognosis stratification of GC. Wang et al. (2020) reported that FLT1 promoted GC peritoneal metastasis through the p-ERK/p-JNK pathway. EGF mediates the activation of Rab35 by DENND1A to regulate GC invasion and migration (Ye et al., 2018). A retrospective study found that COL5A2 was a potential risk factor for prognosis in GC patients (*P* < 0.001, HR = 18.834) (Ding et al., 2021). The above genes have been confirmed to be important participants in the development of GC, and we are also the first to find that they are important markers for GC stratification from a holistic perspective. We then included FLT1, EGF, and COL5A2 with M2 macrophages in the LASSO Cox regression model to establish a prognostic signature to evaluate overall survival. Numerous prognostic signatures have been reported in the past (Zhu et al., 2016; Jiang et al., 2019; Zhao et al., 2019), but these signatures are used to evaluate the prognosis from a certain perspective. It is the first time that we have combined transcriptomics with immune cells to predict the overall survival of GC. We believe that the signature established from a multi-omics perspective provides a new reference for clinical guidance of immunotherapy. However, our research results are mined and verified by bioinformatics methods, and more adequate molecular biological evidence and clinical sample needs to be adopted to prove our viewpoint.

## Conclusion

The influence of Genetic and transcriptional alteration of focal adhesion-related genes on GC patients has been systematically revealed. FAI established based on transcriptomics is an important reference index for evaluating GC prognosis, immune microenvironment, microsatellite instability, TMB, Lauren classification, and sensitive drug molecular screening. FLT1, EGF, and COL5A2 are practicable markers for the identification of GC subtypes. The prognostic signature established together with M2 macrophages provides a new reference for guiding clinical immunotherapy. This study emphasizes the role of focal adhesion-related genes in promoting the progression of GC. Contribute to the development of individualized treatment for GC patients based on the perspective of immunity.

## Data Availability Statement

The datasets presented in this study can be found in online repositories. The names of the repository/repositories and accession number(s) can be found in the article/Supplementary Material.

## Author Contributions

DM, RX, and HC: conceptualization. DM, RX, HC, and SS: data curation. DM, RX, and XC: formal analysis. DM, RX, XC, and DL: data analysis. YH and ZW: funding acquisition. YH, ZW, and CZ: investigation and Project administration. DM, RX, ZW, and CZ: methodology. DM, RX, DL, and SS: resources. DM and RX: original draft and writing—review and editing. All authors contributed to the article and approved the submitted version.

## Conflict of Interest

The authors declare that the research was conducted in the absence of any commercial or financial relationships that could be construed as a potential conflict of interest.

## Publisher’s Note

All claims expressed in this article are solely those of the authors and do not necessarily represent those of their affiliated organizations, or those of the publisher, the editors and the reviewers. Any product that may be evaluated in this article, or claim that may be made by its manufacturer, is not guaranteed or endorsed by the publisher.

## Footnotes

1
https://portal.gdc.cancer.gov/

2
https://www.ncbi.nlm.nih.gov/GEO/

3
http://www.gsea-MSIGDB.org/gsea/MSIGDB/index.jsp

4
https://david.ncifcrf.gov/

5
https://cibersort.stanford.edu/

6
http://www.cancerrxgene.org
