# Supplementary material for: Cross-Talk of Focal Adhesion-Related Gene Defines Prognosis and the Immune Microenvironment in Gastric Cancer
Source: Front Cell Dev Biol. 2021 Oct 1;9:716461. doi: 10.3389/fcell.2021.716461 (PMC8517448; doi:10.3389/fcell.2021.716461)
Supplement: Supplementary file 1 [file Data_Sheet_1.PDF]

Table S1 The focal adhesion-related genes were obtained from c2.cp.kegg.v7.2. symbols of the Molecular Signatures Database.

Table S2 The mutations of 416 genes were related to the prognosis of GC.

Figure S1 Characteristic difference between mutant and non-mutant subtypes. (A) the Dukes-MAC-like stage system was not significantly different between the mutant and non-mutant groups. (B) Differences in mRNAsi of GC samples under different mutation types in the TCGA cohort.

Figure S2 Relationship between CNV alteration of Focal adhesion-related genes and gene expression.

Figure S3 Relationship between FAI and molecular subtype and clinical subtypes. (A) The immune score of the high FAI group was higher than that of the low FAI group in TCGA and GEO cohorts. (B) Chi-square test indicated that the ratio of MSI and low TMB in the low FAI group was higher than that in the high FAI group. (C) The differences between the Epithelial and mesothermal markers in the low-high FAI group were significant. (D) FAI may differentiate between Lauren classification.

Figure S4 FAI was associated with Focal adhesion-related genes and clinical pathological features. (A) There were significant differences in the expression levels of Focal adhesion-related genes between the Low-High FAI groups. (B) The relationship between FAI and clinical pathological features of patients with GC. (C) Univariate cox analysis of 18 Focal adhesion-related genes.

Figure S5 Consistent clustering indicated that relative change of area under CDF curve was the largest when k=2.

Figure S6 Risk prognosis signature to assess the prognosis of GC patients. (A) The ROC curve shows the efficiency of riskscore in assessing the 1-year, 3-year, and 5-year overall survival of GC. (B) Multivariate cox analysis of riskscore and clinicopathological characteristics in the validation cohorts.
