# Supplementary figures and images for: Cross-Talk of Focal Adhesion-Related Gene Defines Prognosis and the Immune Microenvironment in Gastric Cancer
Source: Front Cell Dev Biol. 2021 Oct 1;9:716461. doi: 10.3389/fcell.2021.716461 (PMC8517448; doi:10.3389/fcell.2021.716461)

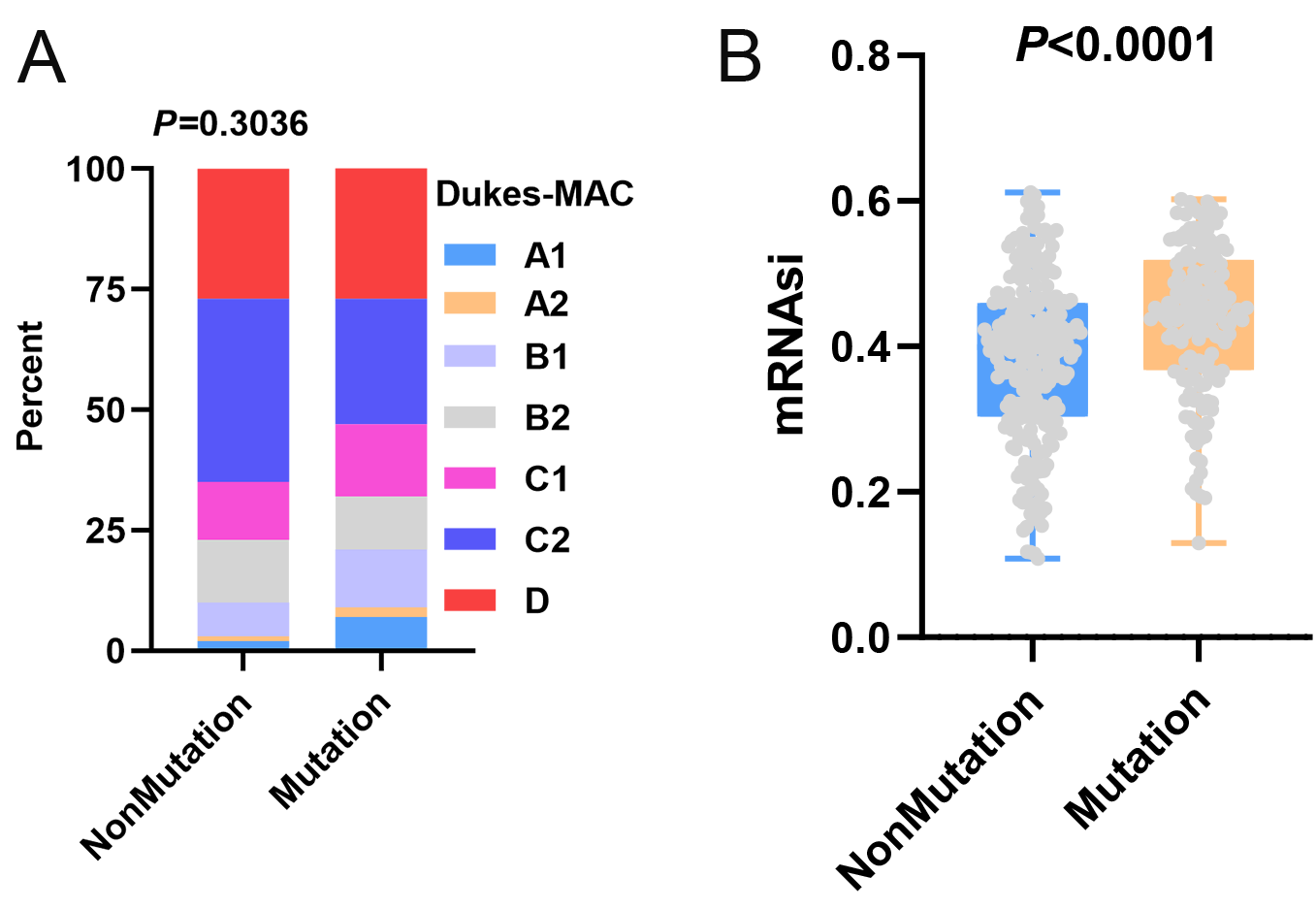

Supplement: Supplementary file 2 [file Image_1.TIF]

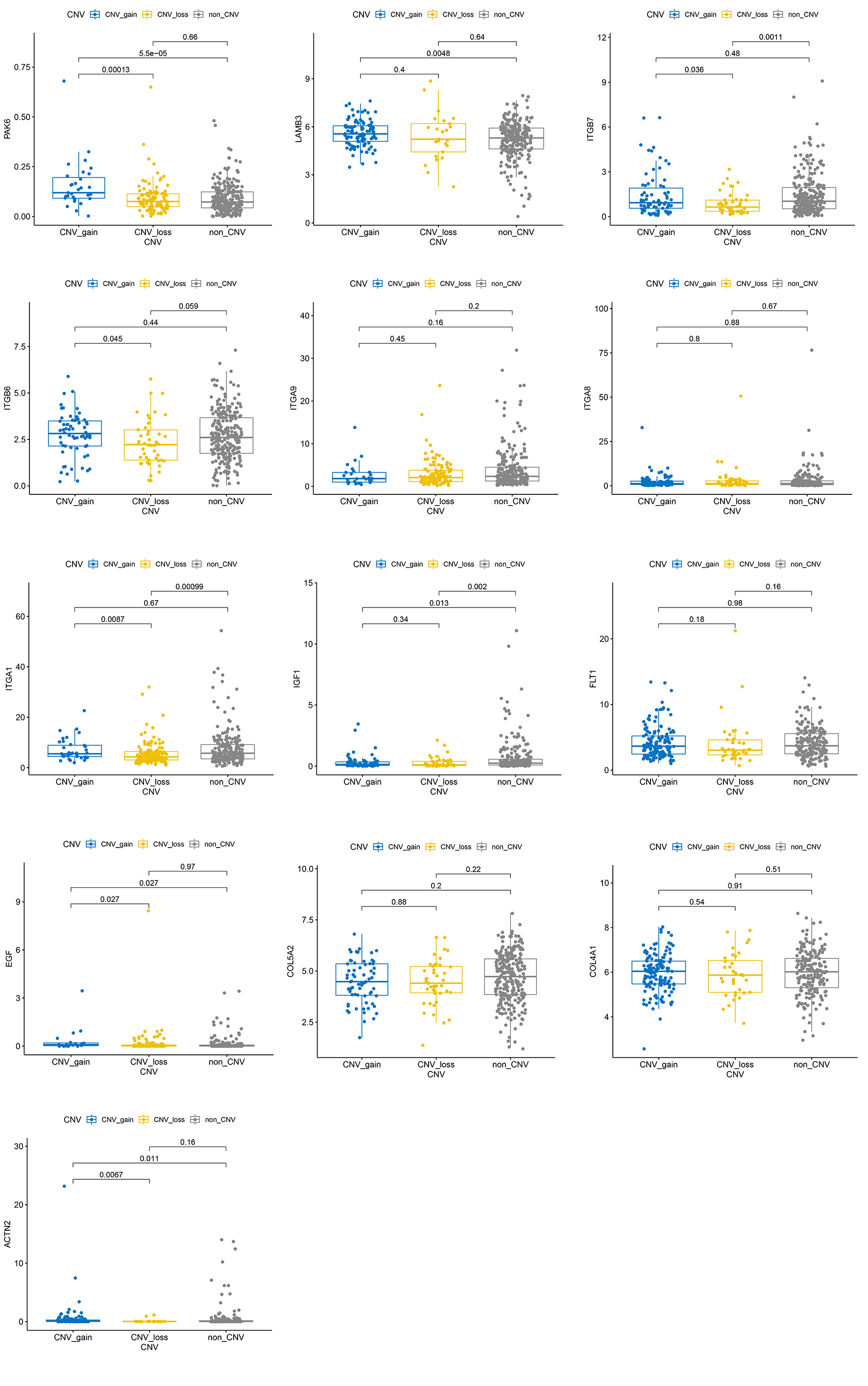

Supplement: Supplementary file 3 [file Image_2.TIF]

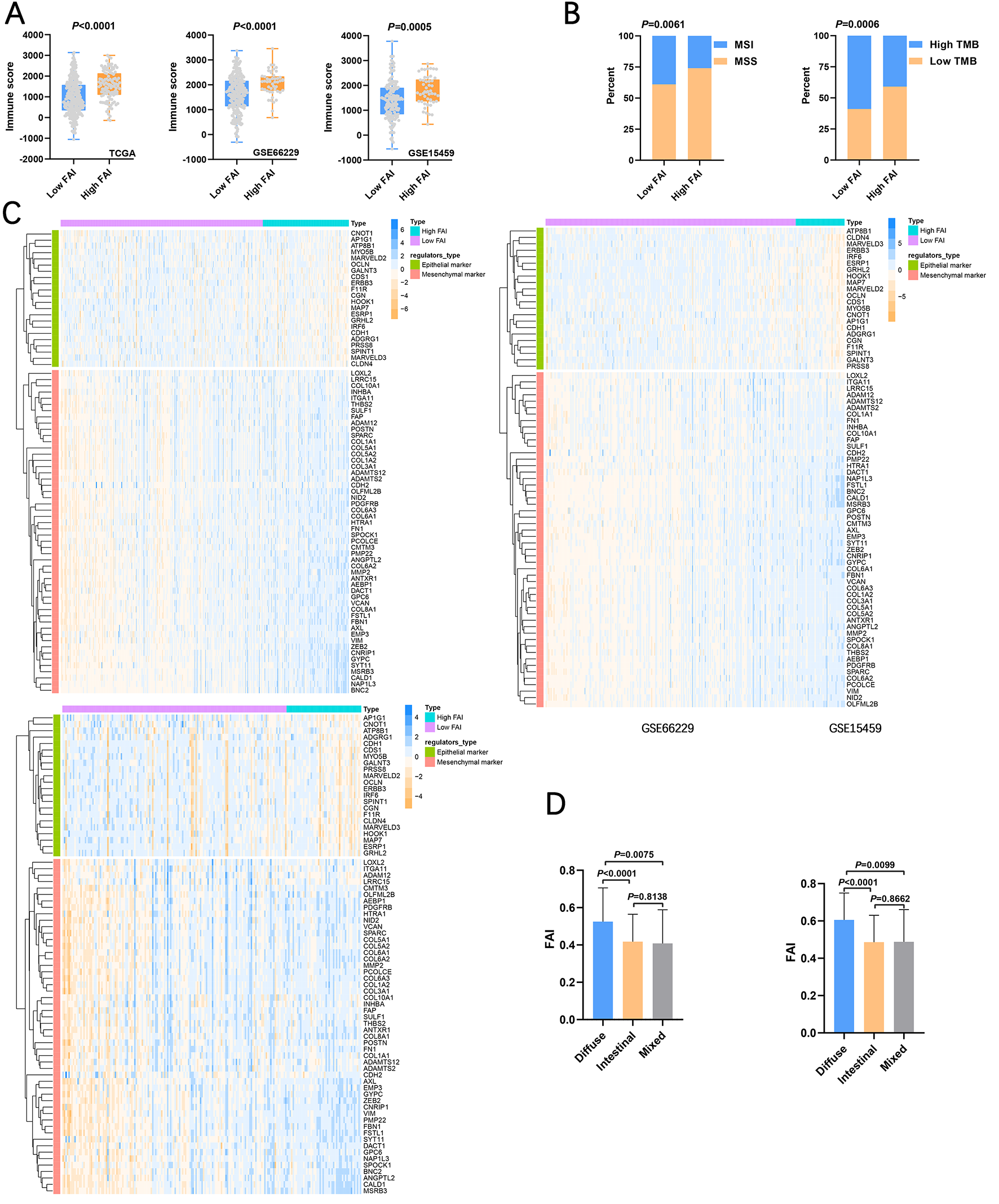

Supplement: Supplementary file 4 [file Image_3.TIF]

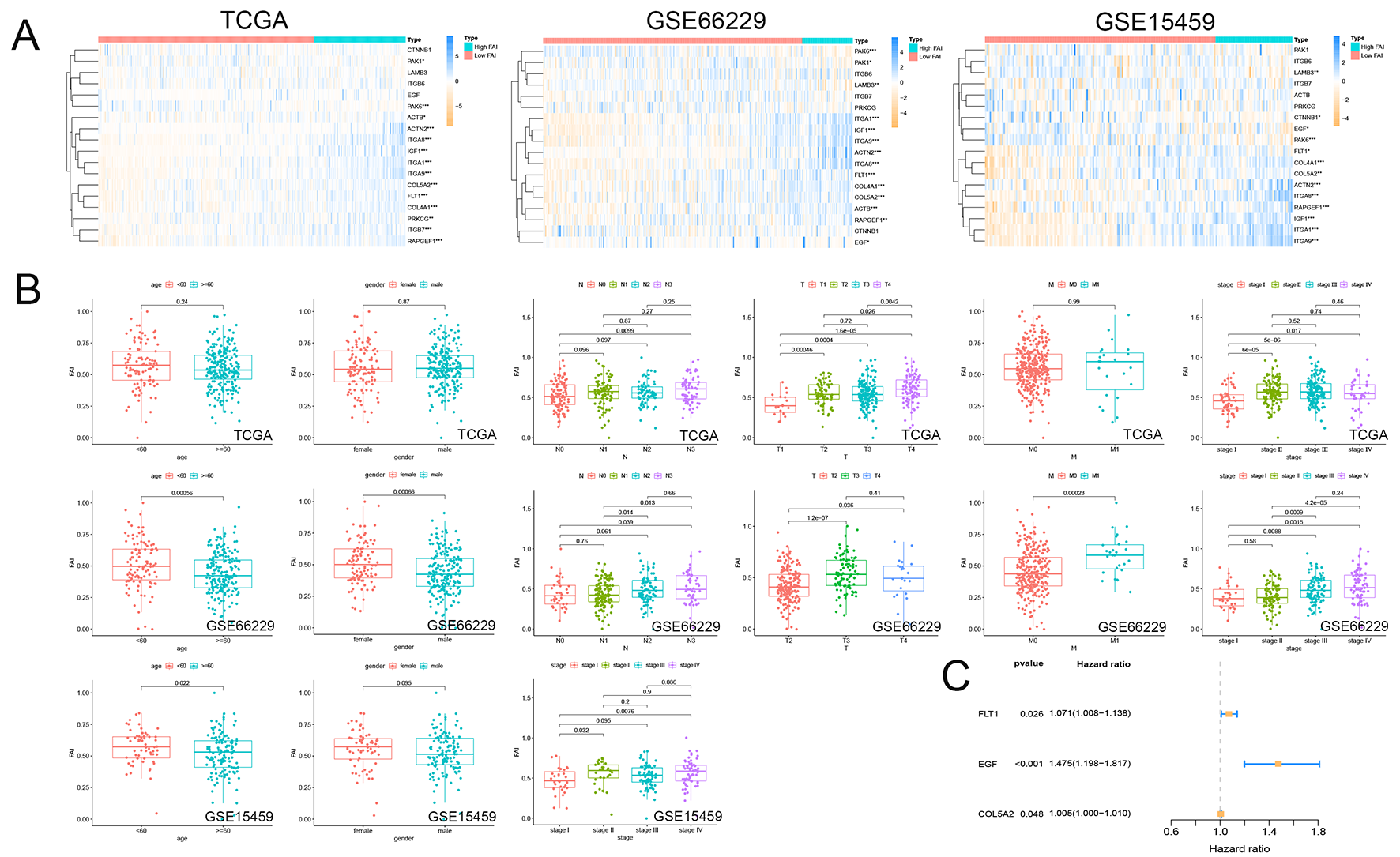

Supplement: Supplementary file 5 [file Image_4.TIF]

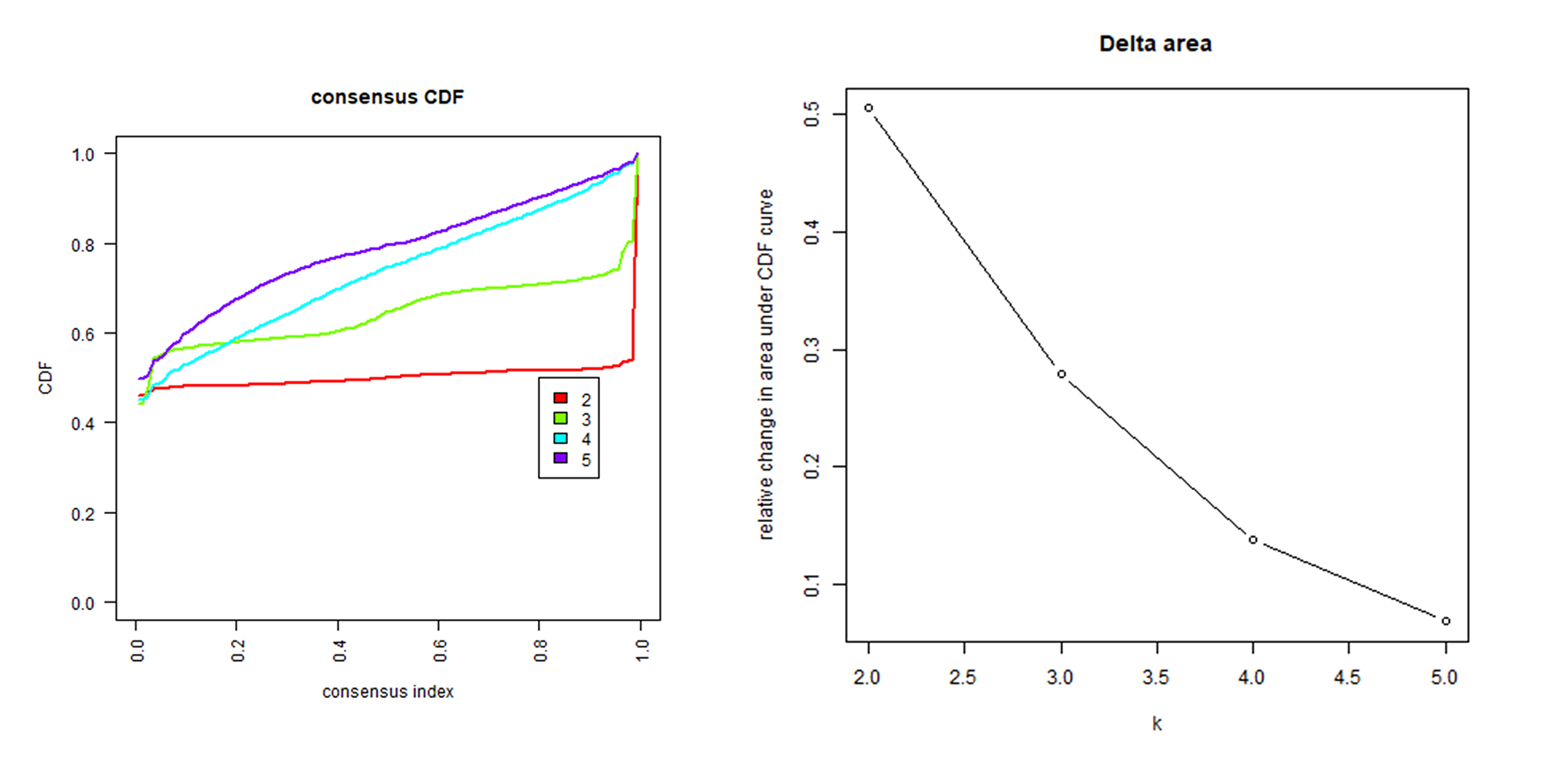

Supplement: Supplementary file 6 [file Image_5.TIF]

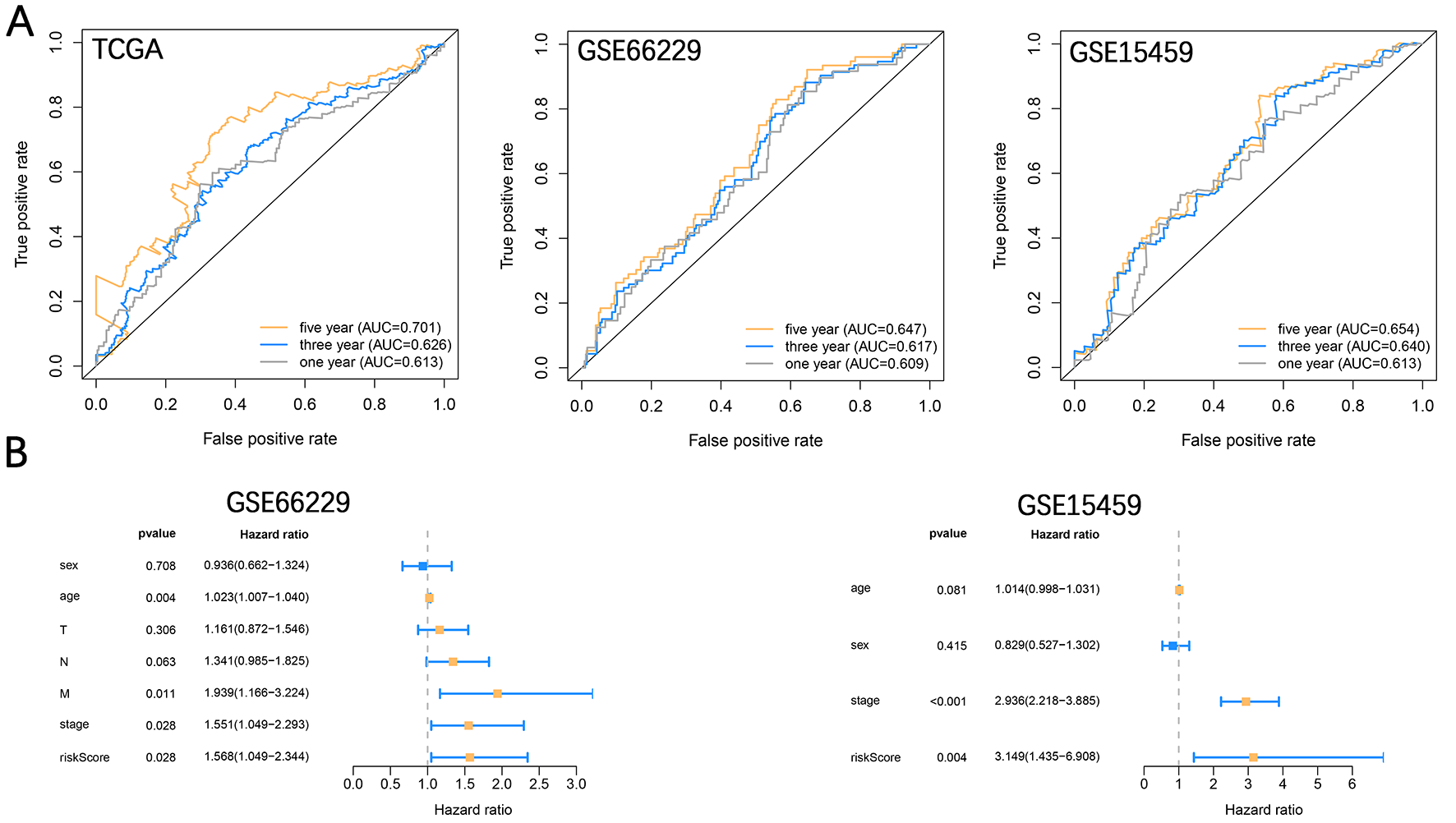

Supplement: Supplementary file 7 [file Image_6.TIF]
